# Synergistic effect of Huyang Yangkun Formula and embryonic stem cells on 4-vinylcyclohexene diepoxide induced premature ovarian insufficiency in mice

**DOI:** 10.1186/s13020-020-00362-6

**Published:** 2020-08-05

**Authors:** Meifang Li, Li Xie, Yang Li, Jian Liu, Guangning Nie, Hongyan Yang

**Affiliations:** 1 Guangzhou University of Chinese Medicine, Guangzhou, Guangdong 510006 China; 2 Department of Gynaecology, The Second Affiliated Hospital of Guangzhou University of Chinese Medicine, Guangzhou, Guangdong 510120 China; 3 Guangdong Provincial Key Laboratory of Clinical Research on Traditional Chinese Medicine Syndrome, Guangzhou, Guangdong 510120 China

**Keywords:** Premature ovarian insufficiency, Traditional Chinese medicine, Huyang Yangkun Formula, Embryonic stem cells, TGF-β/TAK1 signaling pathway

## Abstract

**Background:**

Huyang Yangkun Formula (HYYKF) was developed based on theory of traditional Chinese medicine as well as clinical experience and used to improve ovarian function of premature ovarian insufficiency (POI) patients. Transplantation of embryonic stem cells (ESCs) has great potential in improving POI, and studies have confirmed that traditional Chinese medicine promoted the treatment effect of ESCs. In the present study, we compared the effect of combining HYYKF and ESCs, single HYYKF treatment and single ESCs intervention on POI mice to explore the effect of combination of HYYKF and ESCs in improving ovarian function.

**Methods:**

C57BL/6 mice were used to create a POI model by 15-day intraperitoneal injection of 160 mg/kg of 4-vinylcyclonhexene diepoxide (VCD) and then treated with HYYKF, ESCs transplantation and combination of ESCs and HYYKF. When the treatments were finished, estrus cycle, ovarian follicle counting, serum sex hormone level, and expression of key nodes in the transforming growth factor beta/transforming growth factor beta-activated kinase 1 (TGF-β/TAK1) signaling pathway were determined.

**Results:**

Combination therapy brought down the abnormal estrus cycle rate to 5.26%, significantly lower than that of HYYKF or ESCs alone (30%, 25%, respectively). The numbers of follicles at all levels were increased significantly in the combination ESCs with HYYKF group (P < 0.05), especially that of antral follicles (P < 0.01), which was not increased significantly when HYYKF or ESCs was single used. The level of anti-Mullerian hormone (AMH) was more significantly increased in the combination ESCs with HYYKF group (P < 0.01) than that of HYYKF or ESCs alone (both P < 0.05). The expression of the key nodes TGF-β1, TAK1, JNK, Smad4 and FSHR in the TGF-β/TAK1 pathway were obviously affected in the SCHY group.

**Conclusion:**

Both HYYKF and ESCs improve the ovarian function of POI induced by VCD, and a combination of HYYKF and ESCs has the advantage that they work together to promote follicles developing probably by inhibiting expression of the TGF-β1/TAK1 pathway.

## Background

Premature ovarian insufficiency (POI) is a female reproductive endocrine disease with many complicated causes, leading to ovulation dysfunction, infertility, osteoporosis, and cardiovascular diseases [1]. It is presented in 1–2% of women under 40 years old [2]. The main problem for POI patients is few available follicles in the ovaries. Therefore, the biggest challenge for POI treatment is how to make more follicles developing [3].

As a kind of self-renewing pluripotent cells, stem cells are able to differentiate into cells in a variety of types, and further form specific tissue and organs. Recent studies have shown great potential of embryonic stem cells (ESCs) transplantation in improving the ovarian function [4–6]. ESCs are capable of differentiating into primordial germ cell-like cells and then further forming oocytes in appropriate conditions [7–9]. However, the effect of transplantation treatment is very short and can’t be achieved on ovaries which stayed in pathological state for a long time [10, 11]. It is suggested that reconstruction of ovarian function by stem cells transplantation is related to the ovarian state and the microenvironment where the stem cells are located. In other words, the effect of ESCs transplantation is regulated by the complex microenvironment. Therefore, status of the microenvironment of stem cell colonization is closely linked to the curative effect [12].

Related studies have confirmed that combined with stem cells, traditional Chinese medicine promoted the differentiation of stem cells into endothelial like cells, osteoblasts and other adult cells by regulating differentiation related pathways of Notch and Wnt [13, 14]. Traditional Chinese medicine also promoted the proliferation, homing and tissue repair ability of stem cells. In addition, it has been proved that traditional Chinese medicine could adjust the ovarian microenvironment and play a role in protecting the ovarian function [15–17]. Huyang Yangkun Formula (HYYKF) is mainly used to relieve the symptoms of POI patients. Long-term clinical practice has shown that HYYKF can effectively relieve the symptoms associated with perimenopause of POI patients, especially in psychological and urogenital aspects. It consists of astragali radix, angelicae sinensis radix, rehmanniae radix praeparata, dioscoreae rhizoma, cuscutae semen, epimedii folium, and glehniae radix. The ratio of the seven herbs is 5:1:1:1:1:1:1. Also the formula in our previous study was shown to promote ovarian follicle developing in 4-vinylcyclonhexene diepoxide (VCD)-induced POI rats, and the gene expression of transforming growth factor beta-activated kinase 1 (TAK1) was significantly different between the the control, VCD and VCD + HYYKF groups [18]. TAK1 is an important signaling molecule in the transforming growth factor beta (TGF-β) signaling pathway, and the TGF-β signaling pathway has a wide range of regulating functions by interacting with other pathways and plays a critical role in the physiological and pathological processes of pluripotent stem cells [19]. We hypothesized that HYYKF could enhance the treatment effect of ESCs on POI by regulating the TGF-β/TAK1 pathway. In the study, we used a mouse POI model induced by VCD, to compare the effect of combining HYYKF and ESCs, single HYYKF treatment and single ESCs intervention on POI mice and explored the underlying mechanism.

## Methods

### The animals

Four-week-old female C57BL/6 mice were provided by Beijing Weitong Lihua Laboratory Animal Center (license number: SYXK (Beijing) 2017-0033). The mice were housed under specific-pathogen-free conditions of 20–22 °C and a 12/12 h dark/light cycle. All animals had free access to water and food. The experiment began 1 week after adaptive feeding.

**Fig. 1:**
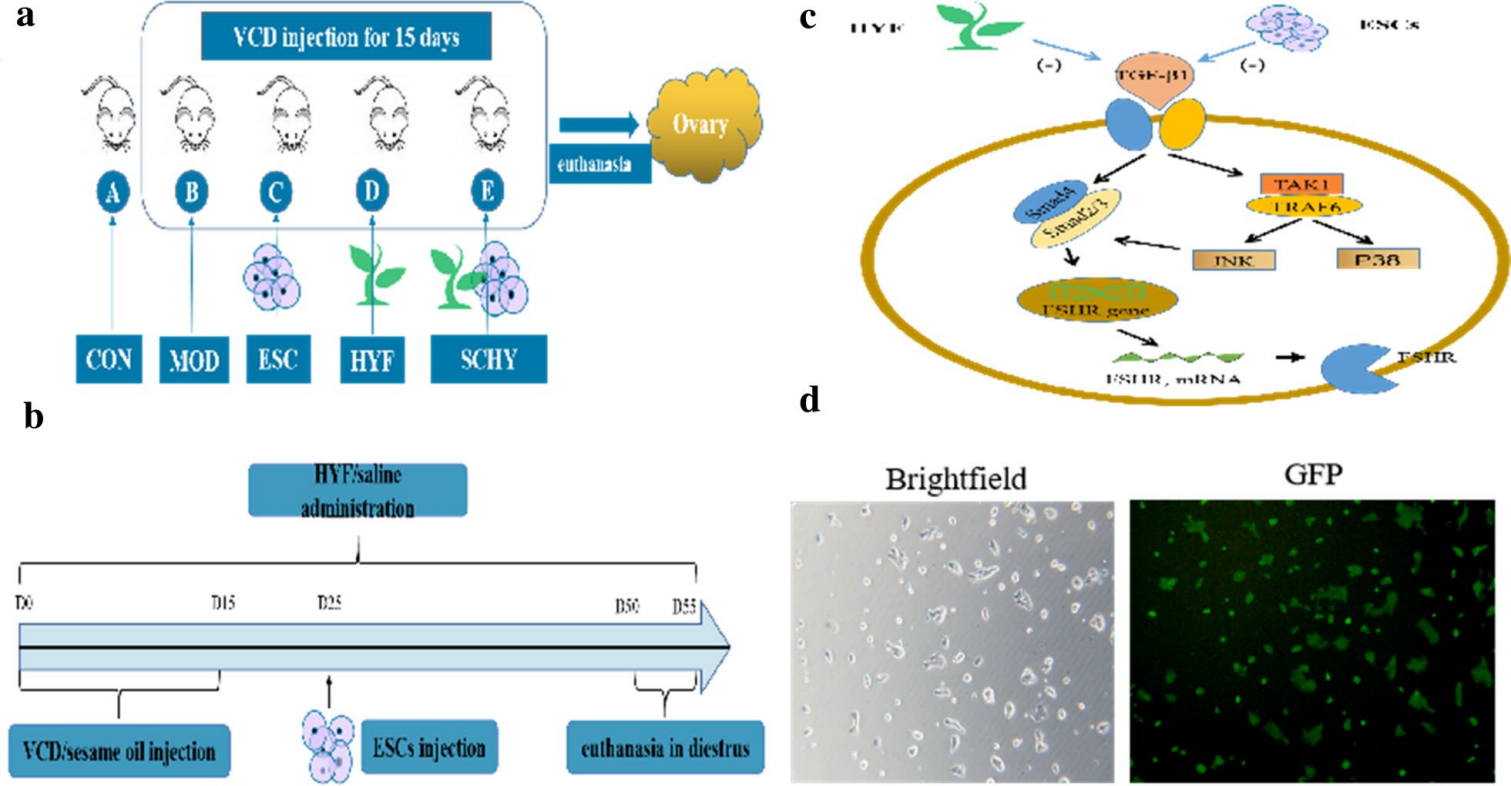
**a** The mice were randomly divided into CON, MOD, HYF, ESC and SCHY groups. **b** From D0 to D15, mice were administered intraperitoneal injection of 160 mg/kg VCD, while mice in the CON group were injected with sesame oil. From D1 to the end, the HYYKF was gavaged in the HYF and SCHY group, while equal volume of saline were gavaged in CON, MOD and ESC group. The mouse ESCs suspension was injected once through the tail vein 10 days at D25 in ESC and SCHY group. **c** The underlying mechanism through TGF-β signaling pathway. **d** The morphology and pluripotency of the ESCs. Brightfield.The morphology of the ESCs under the light microscope. GFP. Most clones were GFP positive, indicating the ESCs were pluripotent and undifferentiated

The mice were randomly divided into control group (CON), model group (MOD), Huyang Yangkun Formula group (HYF), embryonic stem cell group (ESC), and embryonic stem cell combined with Huyang Yangkun Formula group (SCHY) (Fig. 1a). The number of mice in each group is 20. The model was completed by 15-day intraperitoneal injection of 160 mg/kg of VCD diluted in sesame oil, and the control group was injected with sesame oil of the same volume as the model group. The HYYKF was gavaged from the first day of the experiment (D1) in the HYF and SCHY groups. Equal volumes of saline were gavaged in the CON, MOD, and ESC groups. Gavage lasted for 50 days in all groups. The mouse ESCs suspension was injected once through the tail vein 10 days after the modeling (D25) in the ESC and SCHY groups (Fig. 1b). The injection volume is 150 μL, containing 6 × 10^5^ ESCs.

The study was approved by the Guangdong Hospital of Traditional Chinese Medicine Ethics Committees and was carried out in accordance with the NIH Guide for the Care and Use of Laboratory Animals (the euthanasia and disposal of carcasses were in accordance with the guidelines of this guide).

### Pluripotency detection of the ESCs

The ESCs in our study were ESGRO Complete C57BL/6 Mouse Embryonic Stem Cell Line provided by Merck Millipore (Germany, Lot#: SF-CMTI-2). Alkaline phosphatase (AP) was used as a hallmark to detect the pluripotency of the ESCs. High levels of AP were expressed in undifferentiated embryonic stem cells. AP live solution was diluted with ESGRO serum-free base medium in a 1:500 ratio as AP staining solution. The medium in the 24-well plates was discarded, and the cells were rinsed twice using 500 μL of ESGRO serum-free base medium preheated to 37 °C, 3 min each time. Then the cells were resuspended with 200 μL of AP staining fluid and incubated at 37 °C in a 7.5% CO_2_ incubator for 30 min. After then the AP staining fluid was discarded and the cells were rinsed twice again. The medium was added to keep the cells moist, and the staining results were observed under a fluorescence microscope.

### Preparation of HYYKF extract

The composition of HYYKF are astragalus, Angelica sinensis, and other traditional Chinese medicine. The medicine was decocted twice with 8 to 10 times volume of water. Final concentration of the decoction was 1.6683 kg/L. Using high-performance liquid chromatography (HPLC) and ultra-high-performance liquid chromatography-mass spectrometry (UPLC-MS) analysis, it was found that the main active constituents in HYYKF include campanulin, ferulic acid, and icariin and so on [18].

### Analysis of estrous cycle

The estrous cycle analysis was completed with smears of vaginal cells performed at a fixed time every day. The mice vagina was irrigated softly with 10–20 μl of normal saline. The irrigating droplets were collected on a glass slide and then placed horizontally to air dry. After natural air drying, the smears were stained with hematoxylin-eosin (HE). The stained smears were observed under a light microscope and was kept wet during the observation to ensure normal cell morphology. The estrous cycle stage was defined according to cell morphology [20].

### Preparation of pathological sections

After the mice were euthanized (D50-D55), bilateral ovaries, one kidney, and part of the liver blood of each mouse were harvested. Then one of the ovaries, together with liver and kidney, was placed in 4% paraformaldehyde solution to make pathological sections. The tissues were placed in 4% paraformaldehyde solution and soaked overnight at 4 °C. After dehydration and embedding, continuous sections were made with a thickness of 5 μm. HE method was used for staining. Numbers of follicles were determined on every ninth section until the whole ovary was evaluated. The stained sections were observed with a light microscope, and photos were taken.

### Classification and counting of ovarian follicles

The follicles were divided into primordial, preantral, and antral follicles. Primordial follicles were defined as only a single layer of flattened granular cells surrounding the oocytes; preantral follicles were defined as oocytes beginning to develop with surrounding layers of cubic granular cells and the appearance of follicle membrane cells; antral follicles were defined as oocytes growing larger and follicle fluid forming, pushing the oocytes and surrounding granulosa cells to one side. The numbers of primordial, preantral, and antral follicles were recorded, including the total numbers of follicles.

### Detection of sex hormone in serum

To explore the endocrine changes in mice, we adopted the method of enzyme-linked immunosorbent assay (ELISA) to analyze hormone changes in serum. The ELISA kits were purchased from CUSABIO (Lot: CSB-E13156m, CSB-E06871m, CSB-E05109m) to detect serum follicle-stimulating hormone (FSH), anti-Mullerian hormone (AMH), and estradiol (E2) levels in mice. The operations were carried out according to the manufacturer’s instructions.

### Western blotting (WB) analysis

Protein was extracted from the rats’ ovaries using RIPA (radioimmunoprecipitation assay) lysis buffer and was quantified by using a BCA protein assay kit (Thermo Fisher, SE253117A). Sodium dodecyl sulfate polyacrylamide gel electrophoresis (SDS-PAGE) was performed with 10% separation glue. Then the protein on the gel was transferred to polyvinylidene difluoride (PVDF) membrane under the condition of constant 300 mA. 5% skimmed milk dissolved in Tris-buffered saline with Tween (TBST) was used to block the PVDF membrane after transmembrane. The primary antibody was incubated overnight at 4 °C, and the secondary antibody was incubated for 1 h at room temperature. After that, an ECL kit was used to determine chemiluminescence.

The antibodies were TGF-Beta1 Antibody (Proteintech, 21898-1-AP), FSHR Antibody (Proteintech, 22665-1-AP), Anti-Smad4 (Abcam, ab40759), JNK (Phospho-Thr183/Thr183/Thr221) Rabbit pAb (ZENBIO, 384799), JNK Antibody (Proteintech, 10023-1-AP), Phospho-Smad2 (Ser467) Rabbit pAb (ZENBIO, 310079), Smad2/3 Rabbit pAb (ZENBIO, 382472), Phospho-Smad3 (Ser423/425) Rabbit pAb (ZENBIO, 382919), Smad3 Rabbit pAb (ZENBIO, 382774), TAK1 (Phospho-Ser439) Rabbit pAb(ZENBIO, 385849), TAK1 (3G1) Mouse mAb (ZENBIO, 200993), TRAF6 Rabbit pAb (ZENBIO, 385965), p38 MAPK (CST, 8690S), Phospho-p38 MAPK (CST, 4631S), GAPDH Antibody (Boster, BA2913), HRP-linked Antibody (CST, 7074).

### Quantitative real-time polymerase chain reaction (q-PCR) analysis

Critical factors of the TGF-β family were chosen to detect the mRNA expression. Total RNA was extracted from the rats’ ovaries using TRIZOL reagent (ThermoFisher, 15596-026) and transcribed reservedly to cDNA with the GoScript Reverse Transcription System (Promega, A5001). The cDNA was amplified following the instructions of SuperReal PreMix Plus (SYBR Green) under the condition of an initial step at 95 °C (3 min), followed by 40 cycles of denaturation at 95 °C (10 s), and annealing at 60 °C (30 s). The primer sequences are listed in Table 1.

**Table 1:** Sequences of the primers used in this study

Primer	Sequences (5′ to 3′)
Mouse-TAK1	
Forward	CGGATGAGCCGTTACAGTATC
Reverse	ACTCCAAGCGTTTAATAGTGTCG
Mouse-TRAF6	
Forward	AAAGCGAGAGATTCTTTCCCTG
Reverse	ACTGGGGACAATTCACTAGAGC
Mouse-TAB1	
Forward	TCCAACCGCAGCTACTCTG
Reverse	CCCGTACAGGAAGCAGTTATTTT
Mouse-TAB2	
Forward	CATGACCTGCGACAAAAATTCC
Reverse	TGATTGCGTAGACCAGAAATTCC
Mouse-SMAD2	
Forward	ATGTCGTCCATCTTGCCATTC
Reverse	AACCGTCCTGTTTTCTTTAGCTT
Mouse-SMAD3	
Forward	CACGCAGAACGTGAACACC
Reverse	GGCAGTAGATAACGTGAGGGA
Mouse-SMAD4	
Forward	ACACCAACAAGTAACGATGCC
Reverse	GCAAAGGTTTCACTTTCCCCA
Mouse-JNK	
Forward	AGCAGAAGCAAACGTGACAAC
Reverse	GCTGCACACACTATTCCTTGAG
Mouse-P38	
Forward	TGACCCTTATGACCAGTCCTTT
Reverse	GTCAGGCTCTTCCACTCATCTAT
Mouse-βactin	
Forward	GGCTGTATTCCCCTCCATCG
Reverse	CCAGTTGGTAACAATGCCATGT

### Immunohistochemistry (IHC)

The sections were stained by IHC to detect the expression of factors in the TGF-β family. After the antigen was repaired, primary antibodies were added at room temperature for 20 min and then secondary antibodies for 10 min, avoiding light. Diaminobenzidine (DAB) was used for staining, and neutral gum was used to seal pieces. The pieces were observed under light microscope, and 3 fields were collected from each section. ImagePro Plus software was adopted to calculate the average optical density. The antibodies were TGF-Beta1 antibody (Proteintech, 21898-1-AP), anti-TAK1 (Rabbit Monoclonal) (Abcam, ab109526), JNK antibody (Rabbit Polyclonal) (Proteintech, 10023-1-AP), FSHR antibody(Proteintech, 22665-1-AP), Anti-Smad4 (Abcam, ab40759), and a high-efficiency immunohistochemical secondary antibody kit (Absin, abs957).

### Statistical analysis

The data is presented as mean ± standard deviation (x ± s). Statistical analysis was conducted using SPSS version 23.0 statistical analysis software. The Kruskal–Wallis H test and one-way analysis of variance (ANOVA) was used for multiple comparisons, and P < 0.05 was considered statistically significant.

## Results

### The ESCs were in normal morphology and being pluripotent

Under the light microscope, the ESCs grew adherently in the serum-free medium and formed a subspheroidal clone, with clear cloning boundary and less differentiated cells at the edge. The microscopic morphology is shown in Fig. 1d Brightfield. AP is a hallmark of pluripotent stem cells, AP-positive cells were green fluorescent protein (GFP)-positive, and AP-negative cells were GFP-negative. Microscopically, most clones were GFP-positive, indicating that the cultured mouse ESCs were pluripotent and undifferentiated (Fig. 1d GFP).

### The intervention of VCD severely disrupted the estrus cycle

**Fig. 2:**
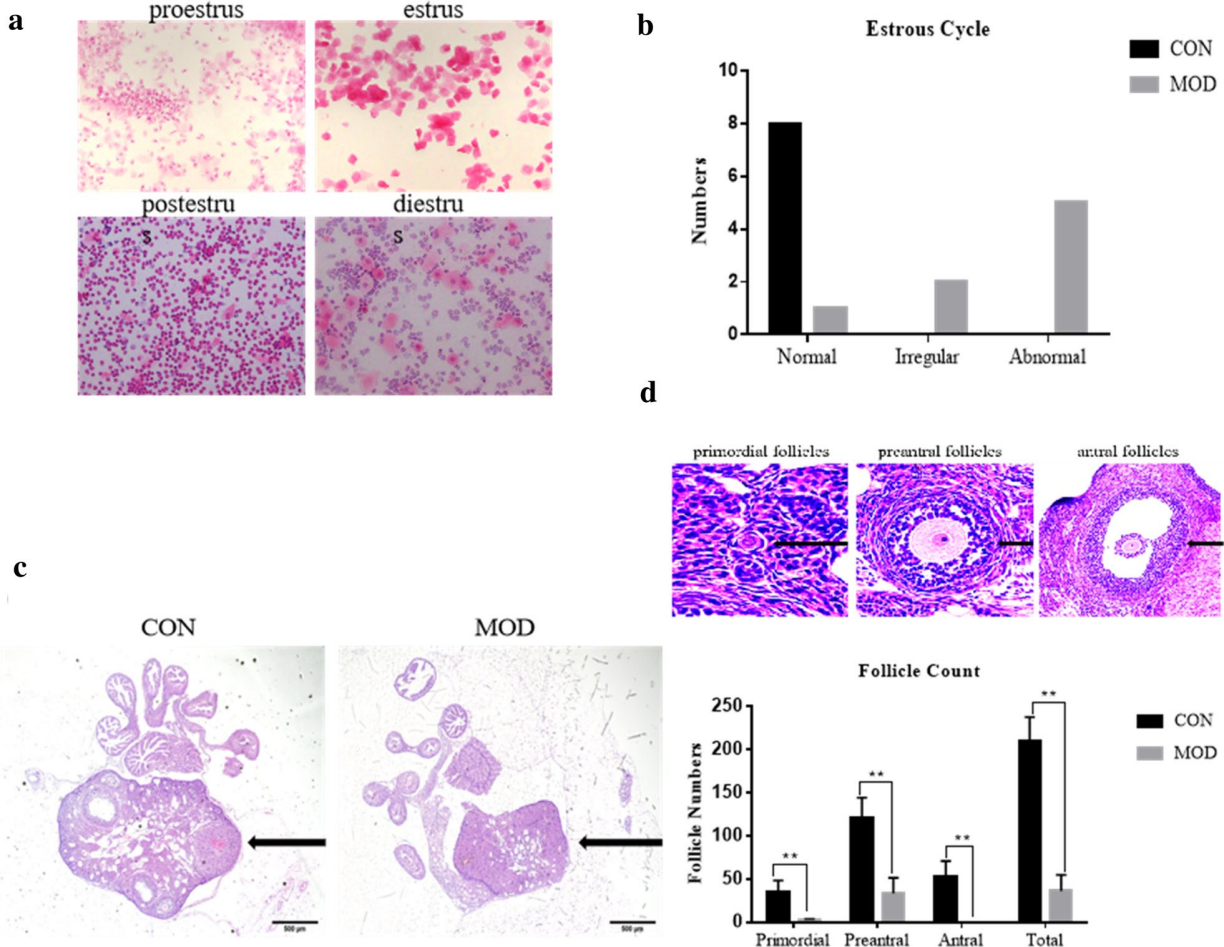
The POI model of mice was created successfully. **a** Estrus cycle monitoring: a.proestrus b.estrus c.postestrus d.diestrus. **b** The estrus cycle of most mice disordered after modeling. **c** Changes in ovarian morphology before and after modeling. Ovaries in CON group and ovaries in MOD group were shown as presented. **d** The numbers of follicles at all levels was decreased in the MOD group compared with the CON group

A normal estrus cycle is an ongoing process of changes with no clear boundaries and usually lasts for 4 to 5 days (Fig. 2a). We defined the estrus cycle as irregular when there was prolonged estrus period or prolonged diestrus period, and as abnormal when there was an estrus interval lasting for 10 days or more. The presentation of the estrus cycle showed that all mice in the CON group were in the normal cycle, while in the MOD group, 1 was in normal, 2 in irregular, and 5 in abnormal estrus (Fig. 2b). This meant that the normal rate was 100% in the CON group, while it was 12.5% in the MOD group, with a significant difference (P = 0.001, P < 0.05), indicating that the estrus cycle of mice was severely disrupted after modeling (Table 2).

**Table 2:** Number of rats in normal, abnormal, and irregular estrus cycle before and after modeling

Group	Normal	Irregular	Abnormal	n	Normal rate %
CON	8	0	0	8	100
MOD	1	2	5	8	12.50
Total	9	2	5	16	56.25

Z =  − 3.303, P = 0.001, P < 0.01

### The intervention of VCD significantly reduced the numbers of follicles

After the injection of VCD, the ovary morphology of mice changed obviously (Fig. 2c). In the CON group, follicles at all levels were observed, and there were large antral follicles with typical follicular morphology and abundant ovarian blood vessels. After VCD modeling, there were few follicles, with more atretic follicles remaining, and the blood supply decreased compared with the CON group. By counting the follicle numbers at each stage, the results showed that the number of follicles at all levels was decreased in the model group, and there was significant difference between the two groups (P = 0.000, P < 0.05) (Fig. 2d).

### The interventions used in this study did not show hepatorenal toxicity

**Fig. 3:**
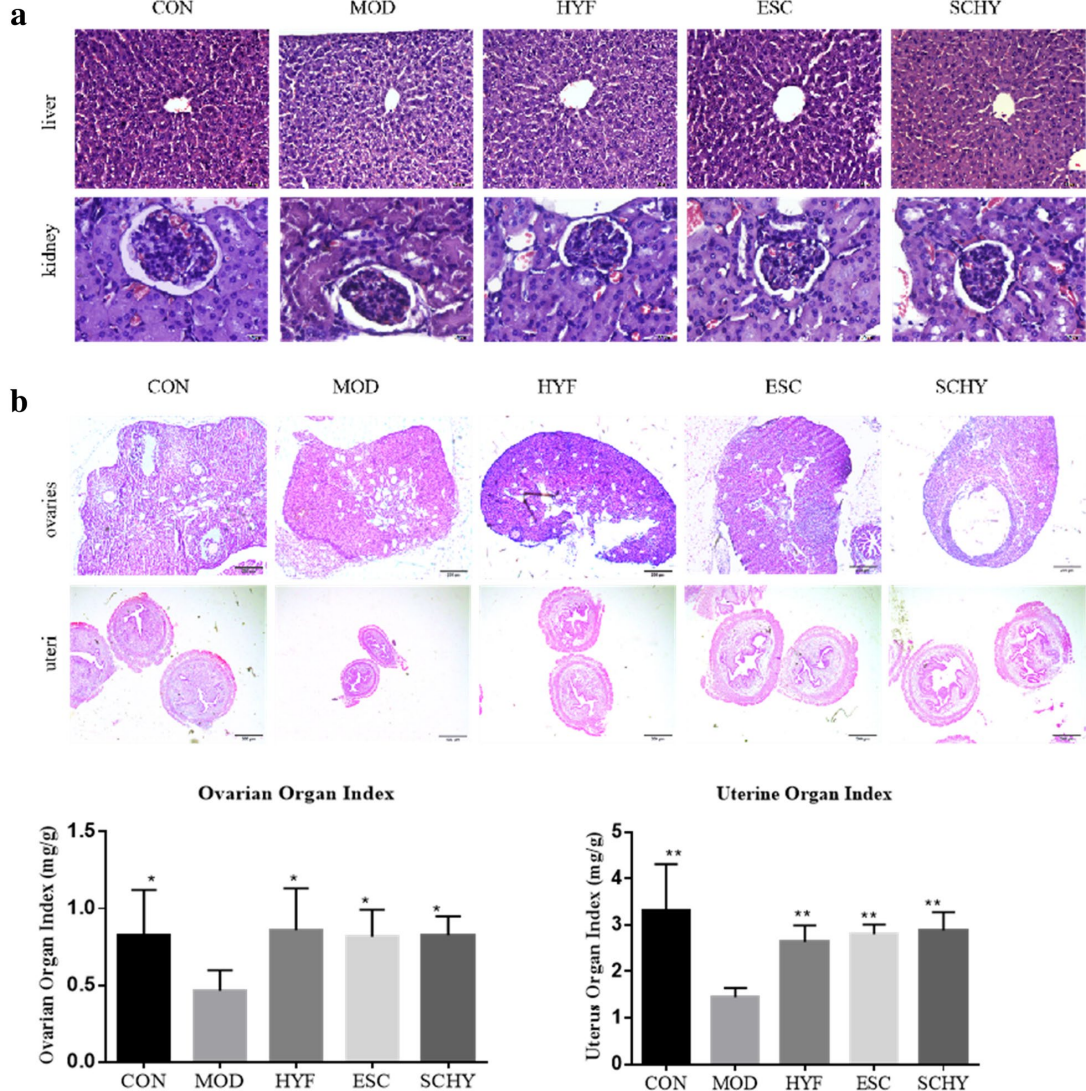
**a** The pathological changes of liver and kidney tissues in each group **b** The organ index of ovaries and uterus increased significantly in the treatment groups. *P < 0.05 **P < 0.01, compared with the MOD group

The pathological changes in the liver and kidney tissues in each group were observed under the light microscope, presenting normal structure and morphology (Fig. 3a). The structure of cells in the liver tissue was normal, the arrangement of cords was regular, the lobule was clearly demarcated, and there was no pathological change in the tissue. Renal cells were also found to be in order. The results indicated that none of the interventions in the study damaged the liver and kidney tissues.

### The organ index of ovaries and uteri increased significantly in the treatment groups

The ovaries and uteri of mice in each group were weighed to calculate organ index. Compared with the CON group, the organ index of ovaries and uteri in the MOD group decreased (P < 0.01 and P < 0.05, respectively), which indicated that VCD had a damaging effect on both ovaries and uteri. Compared with the MOD group, the organ index of ovaries and uteri all increased significantly in the HYF, ESC, and SCHY groups (P < 0.05) (Fig. 3b), indicating that these treatments had a protective effect on both ovary and uterus. There was no significant difference between the HYF, ESC, and SCHY groups (P > 0.05).

### The treatments improved the disordered estrus cycle caused by VCD

**Fig. 4:**
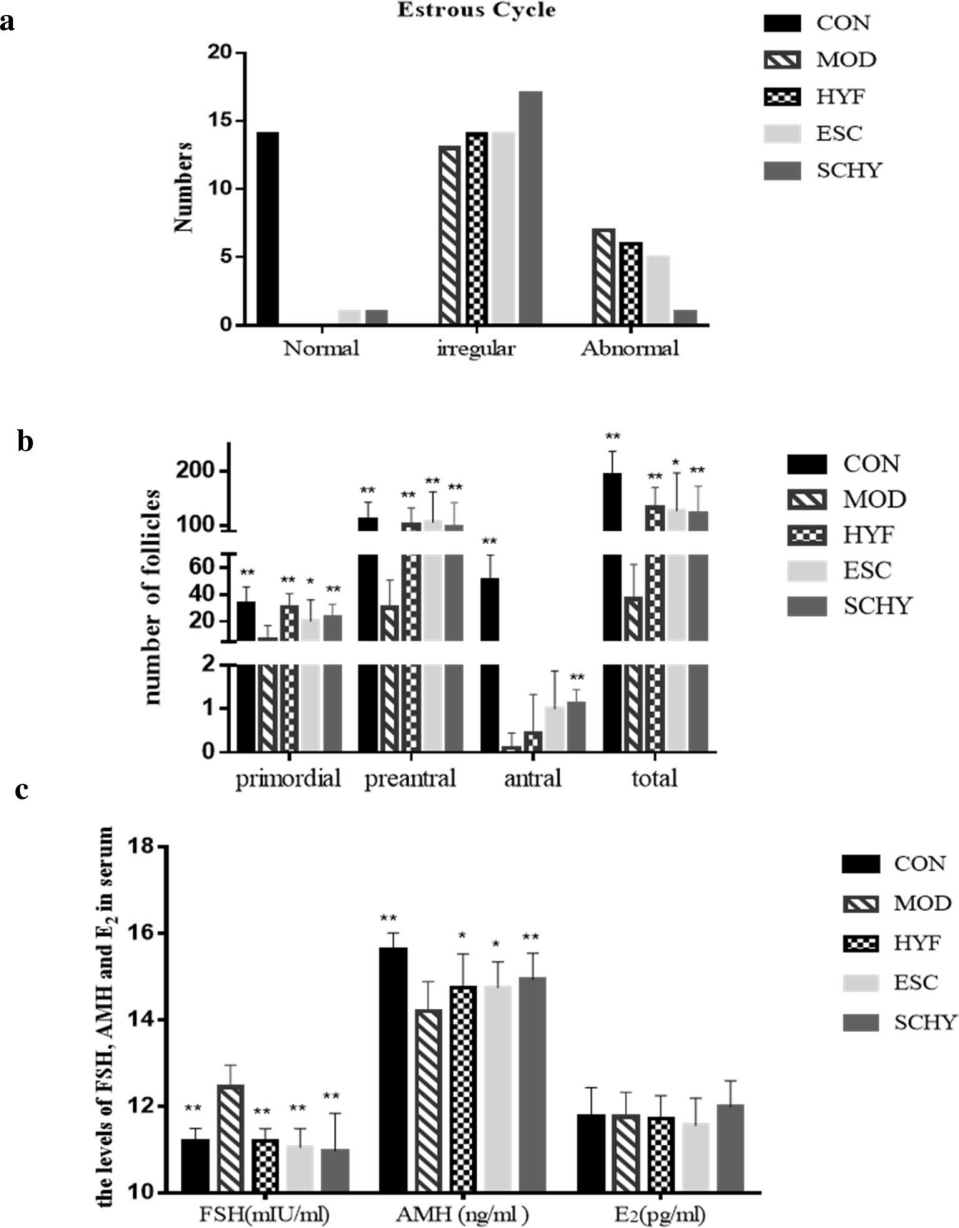
**a** The number of mice in normal, irregular, and abnormal estrus cycle in each group; **b** The number of primordial, preantral and antral follicles and tatal number in each group; **c** The level of FSH, AMH and E2 in serum in each group.*P < 0.05 **P < 0.01, compared with the MOD group

The number of mice in normal, irregular, and abnormal estrus cycles in each group was recorded (Fig. 4a). The rate of normal estrus cycle was 100% in the CON group, 0% both in the MOD and HYF groups, and 5.00% and 5.26% in the ESC and SCHY groups (Table 3). The rate of irregular estrus cycle was 0% in the CON group, 65% in the MOD group, 70% both in the HYF and ESC groups, and 89.47% in the SCHY group. The rate of abnormal estrus cycle was highest (35%) in the model group, lower in the HYF and ESC groups (30% and 25%, respectively), while it was lowest (5.26%) in the SCHY group (χ^2^ = 27.704, P = 0.000, P < 0.01).

**Table 3:** Number and rate of rats in normal, abnormal, and irregular estrus cycle in each group

Group	Normal	Irregular	Abnormal	Total	Normal rate %	Irregular rate %	Abnormal rate %
CON	14	0	0	14	100.00	0.00	0.00
MOD	0	13	7	20	0.00	65.00	35.00
HYF	0	14	6	20	0.00	70.00	30.00
ESC	1	14	5	20	5.00	70.00	25.00
SCHY	1	17	1	19	5.26	89.47	5.26
Total	16	58	19	93	17.20	62.37	20.43

χ^2 ^= 27.704, P = 0.000, P < 0.01

### The numbers of follicles were increased in the treatment groups

Compared with the CON group, the numbers of follicles in the MOD group at all stages were decreased significantly (P < 0.01). And compared with the MOD group, the numbers of follicles at all stages were increased significantly in the HYF, ESC, and SCHY groups (P < 0.05), except that of antral follicles. However, in the SCHY group the numbers of antral follicles increased significantly (P < 0.01), indicating that the combination treatment may have a long-term protective effect on follicle development (Fig. 4b).

### The treatments improved the secretion of FSH, AMH, and E_2_

Serum was separated to detect sex hormone levels (Fig. 4c). The level of FSH in the MOD group was significantly higher than that in the CON group (P < 0.01), indicating that VCD caused a state of high gonadotropin. The levels of FSH in the HYF, ESC,and SCHY groups were relatively lower than that in the MOD group (P < 0.01), showing that these three interventions alleviated the ovarian state of high gonadotropin.

The serum AMH level decreased obviously after the intervention of VCD (P < 0.01), suggesting that VCD had a serious killing effect on the preantral follicles. Compared with the MOD group, the levels in the HYF, ESC, and SCHY groups were all increased. Among them, the difference between the HYF and MOD, ESC and MOD groups were both significant (P < 0.05), while that between the SCHY and MOD group was more significant (P < 0.01), indicating that the combination intervention was superior to single intervention.

There was no significant difference of the E_2_ level between the groups (P > 0.05), suggesting that the ovarian function not totally failed at 50–55 days after modeling. The level of E_2_ increased as feedback to the increase of FSH, although the ovaries had begun to age.

### The expression of the TGF-β1/Smad2/3/Smad4/FSHR signaling pathway was regulated by the treatments

**Fig. 5:**
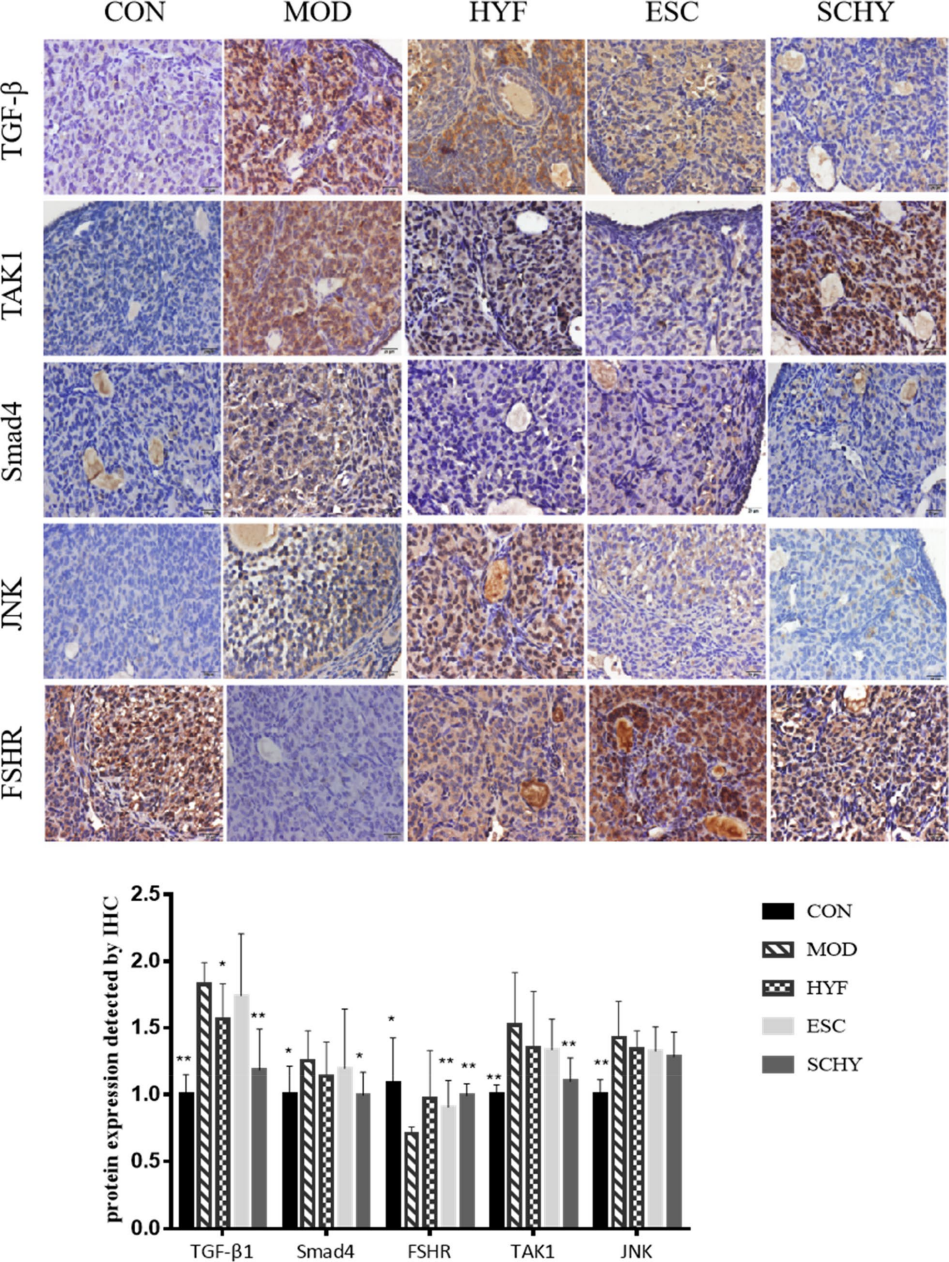
Immunohistochemistry (IHC) analysis for TGFβ1, TAK1, Smad4, JNK and FSHR in ovaries

**Fig. 6:**
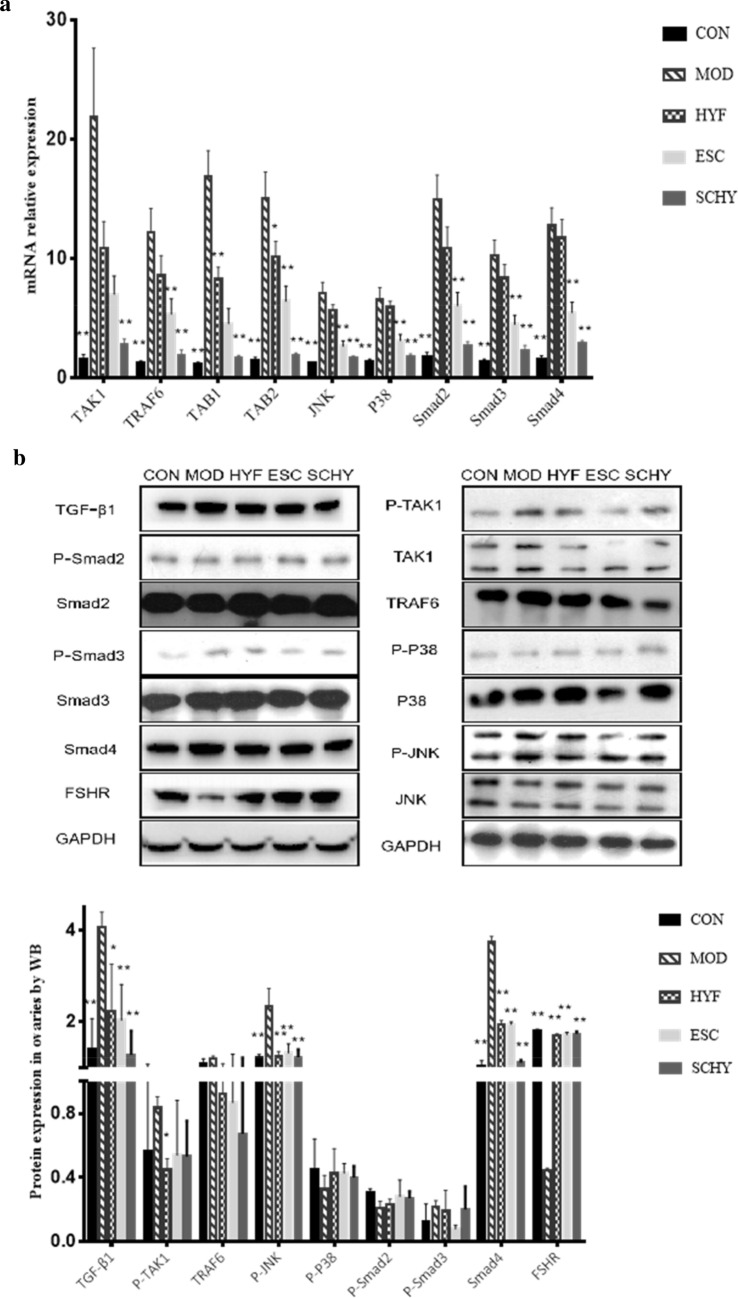
**a** The expression of TAK1, TRAF6, TAB1, TAB2, JNK , P38, Smad2, Smad3 and Smad4 analysied by q-PCR. **b** The expression of TGF-β, TAK1, TRAF6, JNK , P38, Smad2, Smad3, Smad4 and FSHR analysied by WB

We used IHC and WB to analyze the expression of TGF-β1, Smad4, and FSHR protein and WB to analyze the expression of Smad2, Smad3, and their phosphorylated counterparts (Figs. 5, 6b). Compared with the CON group, the expression of TGF-β1 in the MOD group was significantly upregulated (P < 0.01). Then the expression in the HYF and SCHY groups was relatively lower (P < 0.05), compared with the MOD group. There was no difference between the ESC group and the MOD group. We obtained similar results using WB tests. The expression of TGF-β1 was significantly increased by VCD (P < 0.05) and decreased in all the treatment groups (P < 0.05). The expression of Smad4 increased significantly in the MOD group tested by IHC and WB (P < 0.05, P < 0.01, respectively), and then declined in the treatment groups, especially in the SCHY group with statistical difference (P < 0.05, P < 0.01, respectively), while the expression of FSHR was downregulated in the MOD group tested by IHC and WB (P < 0.05, P < 0.01, respectively) and upregulated in the treatment groups, especially in the SCHY group (P < 0.01, P < 0.01, respectively). The change trend of P-Smad3 protein expression was consistent with Smad4, but there was no significant difference between the groups.

The expression of Smad4, Smad2, and Smad3 mRNA showed the same trend. Compared with the CON group, the mRNA expression in the MOD group was increased statistically significantly (P < 0.05). The expression decreased significantly in both the ESC group and the SCHY group (P < 0.05), while there was no statistical difference between the HYF group and the MOD group.

### The expression of the TGF-β1/TAK1/JNK/p38 signaling pathway was regulated by the treatments

IHC was also used to detect the expression of other proteins(Fig. 5). Compared with the CON group, the expression of TAK1 protein in the MOD group was significantly induced (P < 0.01). Compared with the MOD group, the expression in the SCHY group was inhibited (P < 0.01). The expression of JNK protein in all other groups was increased compared with the CON group (P < 0.01). However, there was no significant difference between the treatment groups and the MOD group.

For further exploration, we examined the mRNA expression of TAK1, JNK, and P38, as well as the expression of TAKI related tumor necrosis factor receptor-associated factor 6 (TRAF6), TAK1 binding protein (TAB1), and TAB2 genes by real-time quantitative PCR (Fig. 6a). The expression of TAK1 was significantly upregulated by VCD (P < 0.01). There was no statistical difference between the HYF and MOD groups or the ESC and MOD groups. However, the expression of TAK1 was decreased significantly in the SCHY group (P < 0.01). The expression of TRAF6, TAB1, and TAB2 all decreased significantly in the MOD group and rose significantly in the SCHY group (P < 0.05, P < 0.05, respectively). HYF alone only decreased the expression of TAB1 and TAB2 (P < 0.05, P < 0.01, respectively), while ESC alone only decreased the expression of TRAF6 and TAB1 (P < 0.05, P < 0.01, respectively). Similarly, VCD induced the expression of JNK and P38 (P < 0.01), ESCs alone and combined with HYYKF both inhibited the expression (P < 0.01 and P < 0.01, respectively).

TAK1, JNK, P38, TRAF6 and their phosphorylated counterparts were determined by WB. Consistent with mRNA expression, the expression of P-TAKI and P-JNK protein was up-regulated in the MOD group and down-regulated in the treatment groups. The difference of P-JNK was very significant (P < 0.01). As to the expression of P-P38 and TRAF6, there was no significant difference between the groups.

## Discussion

In the study, we found that both HYYKF and ESCs improved the ovarian function of POI mice induced by VCD from the perspectives of estrus cycle, ovarian and uterine organ index, follicles counting, and serum sex hormone level. Furthermore, the method of combining HYYKF and ESCs (i.e., the SCHY group) showed its advantage over single HYYKF treatment or ESCs intervention to some extent. It was also found that the effect of improvement may work via the TGF-β/TAK1 signaling pathway.

VCD is a by-product produced in the manufacturing process of rubber tires, flame retardants, insecticides, plasticizers, and antioxidants [21]. Intraperitoneal injection of VCD is an ideal modeling method for the study of POI [22]. In the study, VCD significantly disrupted the estrus cycle and decreased the ovarian follicle number (P < 0.01), successfully creating a POI model of mice. Studies have shown that ESCs directly differentiate into female germ cells, thus reversing the depletion of ovarian follicles [23–25]. At the same time, the HYYKF showed its potential in promoting ovarian function [18]. In our study, both HYYKF treatment and ESCs intervention improved the ovarian function of VCD-induced POI in mice, and the combination of HYYKF and ESCs showed an advantage over single HYYKF treatment or ESC intervention in improving the ovarian function. In terms of improving the estrus cycle, although combination therapy does not have an obvious advantage in maintaining normal estrus cycle, the abnormal rate of combination therapy is only 5.26%, significantly lower than that of HYYKF or ESCs alone (30% and 25%, respectively). In promoting development of follicles, combination therapy induced much more follicles to develop into antral follicles. At the same time, the difference of AMH level between the SCHY and CON groups (P < 0.05) was smaller than that between the HYF and CON and ESC and CON groups (both P < 0.01). These data implied that combining ESCs with HYYKF may work better in improving ovarian function.

Then, we attempted to indicate the underlying mechanism. The TGF-β signaling pathway is activated during the differentiation and formation of the early embryonic mesoderm and contributes a lot to the renewal, maintenance, and differentiation of pluripotent stem cells [26, 27]. Apart from the canonical Smad-dependent pathways, the non-canonical pathways involving TAK1 act through its interplay with JNK and p38 signaling pathways to regulate cellular processes. Also, a study revealed an ameliorating effect of chrysin on radiation-induced POI, and the effect worked via the TGF-β/MAPK (mitogen-activate protein kinase) signaling pathway, indicating that the pathways mediated inflammatory and apoptotic signal transduction in POI [28]. Another study confirmed that FSHR expression is downregulated during ovarian follicular atresia, and apoptosis of granulosa cells was mediated through the FSHR signaling pathway activated by TGF-β1 [29]. In granulosa cells of the ovaries, TGF-β1 activates Smad2/Smad3 to phosphorylate, then the activated Smad2/Smad3 form a polymer with Smad4 to promote FSHR expression and regulate follicular development. As a member of the MAPK kinase kinase (MAPKKK) family, TAK1 is a key molecule in Smad-independent the TGF-β signaling pathway [30], acting as the upstream molecule in the JNK/P38 signaling pathway [31]. After being activated by TGF-β1, TAK1 activates JNK and P38 [32], regulating the apoptosis of cells. At the same time, JNK also induces the phosphorylation of Smad2/Smad3, where connects the two branches of TGF-β/Smads/FSHR pathway and TGF-β1/TAK1/JNK/P38 pathway. So the whole TGF-β1/TAK1 pathway plays a vital way in apoptosis of ovarian cells and follicular atresia. In our previous study, the gene expression of TAK1 in rats was obviously affected by VCD and HYYKF, meaning that TAK1 plays an important role in ovarian function. In this study in mice, it was also significantly affected by VCD, and compared with the MOD group, combination therapy had a more significant effect than HYYKF or ESCs alone. The activation of TAK1 relies on its specific binding protein called TAB, as well as TRAF6 [33–36]. Among them, TAB1 mediates the autophosphorylation of TAK1, while TAB2 binds with TRAF6, inducing the activation of TAK1. The tendency of these molecules to change between groups is exactly the same as that of TAK1. These data suggest that factors in the TGF-β1/TAK1 pathway were regulated by HYYKF, ESCs, and their combination (Fig. 1c), especially that the key nodes TGF-β1, TAK1, JNK, Smad4 and FSHR were obviously affected in the SCHY group, suggesting that HYYKF and ESCs worked together to regulate expression of the TGF-β1/TAK1 pathway. And the factors were also regulated in the ESC group, indicating transplantation of ESCs induced secreting some factors, which promote the development of ovarian follicles. This may be another therapeutic mechanism of ESCs besides direct differentiation into body cells.

We created a POI model of mice, monitored the effects of combination of HYYKF and ESCs in improving ovarian function, and found the potential mechanism of the TGF-β1/TAK1 signaling pathway. However, there were several limitations in our present study. The main methods of stem cell transplantation are vein transplantation and local injection transplantation. Venous transfusion is easier to operate compared with local injection, but venous obstruction in the lung affects its homing rates, which decreases the curative effect. Ovaries of mice are small. In particular, the ovaries became smaller and stuck to the surrounding tissue after modeling, making it very difficult to administer local injection. In this study, we failed to carry out local injection successfully. Therefore, an improved local injection technique should be a topic of future studies. In addition, the time and number of transplantation affect the homing effect. The earlier ESCs were transplanted, the higher the homing rate was. In this study, the mouse ESCs suspension was injected once 10 days after the modeling. We did not investigate when and how much the ESCs should be injected. Further studies are required to explore the time and amount of ESCs injection.

## Conclusion

Both HYYKF and ESCs improve the ovarian function of POI induced by VCD, and a combination of HYYKF and ESCs has the advantage that they work together to promote follicles developing probably by inhibiting expression of the TGF-β1/TAK1 pathway.

## Data Availability

The datasets used and/or analyzed during the current study are available from the corresponding author on reasonable request.

## Abbreviations

HYYKF: Huyang Yangkun Formula
POI: Premature ovarian insufficiency
ESCs: Transplantation of embryonic stem cells
VCD: 4-vinylcyclohexene diepoxide
TGF-β: Transforming growth factor beta
TAK1: Transforming growth factor beta-activated kinase 1
AMH: Anti-Müllerian hormone
CON: The control group
MOD: The model group
HYF: The Huyang Yangkun Formula group
ESC: The embryonic stem cell group
SCHY: The embryonic stem cell combined with Huyang Yangkun Formula group
AP: Alkaline phosphatase
HPLC: High-performance liquid chromatography
UPLC-MS: Ultra-high-performance liquid chromatography-mass spectrometry
FSH: Follicle stimulating hormone
E2: Estradiol
ALT: Alanine aminotransferase
AST: Aspartate aminotransferase
BUN: Blood urea nitrogen
Cr: Creatinine
HE: Hematoxylin–eosin
ELISA: Enzyme-linked immunosorbent assay
WB: Western blotting
RIPA: Radioimmunoprecipitation assay
SDS-PAGE: Sodium dodecyl sulfate polyacrylamide gel electrophoresis
PVDF: Polyvinylidene difluoride
TBST: Tris-buffered saline with Tween
q-PCR: Quantitative real-time polymerase chain reaction
IHC: Immunohistochemistry
DAB: Diaminobenzidine
GFP: Green fluorescent protein
ANOVA: One-way analysis of variance
